# Current Advances in Breast Reconstruction

**DOI:** 10.3390/jcm11123328

**Published:** 2022-06-10

**Authors:** Jevan Cevik, David J. Hunter-Smith, Warren M. Rozen

**Affiliations:** 1 Department of Surgery, Peninsula Clinical School, Central Clinical School, Faculty of Medicine, Monash University, 2 Hastings Road, Frankston, VIC 3199, Australia; jevancevik@gmail.com (J.C.); dhuntersmith@me.com (D.J.H.-S.); 2 Plastic and Reconstructive Surgery Group, Peninsula Clinical School, Peninsula Health, Monash University, 2 Hastings Road, Frankston, VIC 3199, Australia

Breast cancer management is multidisciplinary, and while oncologic surgery, adjuvant therapy, and psychological therapies are central to this, breast reconstruction also forms an integral part of management. Women facing breast cancer have numerous options available to them to restore the aesthetics of the breast after surgical resection. The remarkable outcomes that patients are offered in the current day are a result of numerous technological advancements throughout history that have enabled the practice of breast reconstruction to evolve into its modern form. Equally, the future of breast reconstruction will be paved by the achievements realised by the researchers of today.

For decades, breast reconstruction was not widely utilised, with early theories discouraging its use, such as those from the famous surgeon Halsted suggesting that it may increase the likelihood of cancer recurrence or dissemination [1]. However, as the understanding of breast cancer and breast surgery has improved, a growing role for breast reconstruction has emerged to restore the aesthetic appearance of the breast for patients. In 1895, Vincenz Czerny performed the first autologous breast reconstruction, transplanting a lipoma from a patient’s flank [2]. In the years that followed, innovative techniques were based on local myocutaneous flaps. For instance, in 1896, Ignio Tanzini introduced the latissimus dorsi myocutaneous flap for the closure of a mastectomy defect, and in 1906, Louis Ombrédanne performed a breast reconstruction using the pectoralis minor muscle as a mound [3,4]. Following these early techniques, tubed pedicle flap techniques were developed, such as those performed by Sir Harold Gillies in 1942 [5]. However, these early procedures sometimes required multiple operations and transfers and often resulted in unsightly scars for the patient. Hence, this prompted the introduction of prosthetic implant-based reconstructions in the decades that followed, leading to the development of the first silicone implant by Cronin and Gerow in 1963 [6]. It was not until a decade later, when Daniel and Taylor introduced the world to the free flap in 1973, that the modern techniques for breast reconstruction began to emerge [7]. For the first time, patients were given the option to use autologous tissue from a distant site that more accurately resembled natural breast tissue. Much research in this field led to the first abdominal-based free flap performed by Holmstrom in 1979 and to the development of the transverse rectus abdominis myocutaneous (TRAM) flap by Hartrampf three years later [8,9]. Some years later, in 1994, building upon work initiated by Koshima and Soeda, Robert Allen introduced the field of breast surgery to the deep inferior epigastric artery perforator (DIEP) flap [10,11]. Today, the DIEP flap has grown in popularity to become one of the most frequently performed free flaps for autologous breast reconstruction.

The technological advancement in breast reconstructive surgery largely stemmed from a development in the understanding and practice of microsurgery. The first reported cases of end-to-end vascular anastomosis date back to Murphy in 1897 and the triangulation method introduced by Alexis Carrel in 1902 [12,13]. Since then, the field has developed to include super-microsurgery involving the anastomosis of vessels less than 0.8 mm in diameter [14]. Much of the progress of microsurgery occurred in the 1970s. Following the discovery of the free flap by Daniel and Taylor as mentioned previously, an increased understanding of vascular anatomy led to the development of numerous techniques for free-tissue transfer. The introduction of perforator-based flaps such as the DIEP flap offered reduced donor site morbidity by preserving muscle tissue. However, this carried with it a corresponding increase in the difficulty and complexity of performing the procedure leading to challenging dissections. Hence, in response, new technological advancements were required to aid surgeons in performing breast reconstructions. One of the innovations that followed was the introduction of preoperative imaging techniques. Early on, this imaging was dependent on Doppler ultrasound but eventually resulted in the discovery of Computed Tomographic Angiography (CTA) for preoperative planning [15,16]. With the advent of CTA, surgeons were able to develop reconstructions of the vascular anatomy to identify perforators, map their course, and plan flap design prior to the day of surgery. CTA quickly became the gold standard for pre-operative imaging, particularly for DIEP flaps and breast reconstruction [15,16,17,18].

Innovation in the field of breast reconstruction and microsurgery were not limited to preoperative imaging. Since its introduction to the operating room in 1921 by Carl-Olof Nylén, the operating microscope has allowed surgeons to perform increasingly challenging anastomoses with progressively smaller vessels [19]. Furthermore, efficiency in the operating room has improved with the introduction of microvascular anastomotic coupler devices, reducing operative durations and allowing a greater through-put of cases to be performed [20,21,22]. A significant challenge faced by plastic surgeons is insufficient perfusion of the flap, leading to partial or even total flap failure. The traditional method to evaluate perfusion of the flap is based on a clinical assessment performed by the surgeon. However, numerous promising methods for assessing flap perfusion including fluorescence imaging and laser Doppler imaging intraoperatively and postoperatively may offer surgeons a more sensitive method to identify flap compromise [23,24,25].

On the horizon of breast surgery research, techniques in pre-operative planning and imaging are still a topic of much interest. Thermographic imaging, while being a relatively old concept, has recently been adapted for use in breast reconstructive surgery [26]. The use of infrared thermographic cameras as an alternative or adjunct to CTA for the localisation of perforators has been suggested. Additionally, recently studied augmented reality (AR) or three-dimensional printing technology offers the potential to create reconstructions that can be overlaid onto patients to represent the vascular anatomy in real time on the day of surgery [27,28]. Furthermore, the prospect of robotic-assisted plastic surgery in the field of breast reconstruction has been proposed to potentially advance the abilities and capabilities of what surgeons are able to currently achieve [29]. The combination of virtual reality and robotic surgery together may offer even further utility.

Over the last few decades, the field of breast reconstruction has seen many changes in the form of new knowledge and technology that has led to the development of modern practices. What has been mentioned in this editorial is just a fraction of the advances that have changed the face of breast reconstruction not only for surgeons but also, and more importantly, for patients with breast cancer. We hope that this Special Issue of the *Journal of Clinical Medicine* brings together an array of impactful evidence that may contribute to the vast history of innovation in the field of breast reconstruction and, as a result, further enhance the outcomes experienced by patients.

## Data Availability

Not applicable.
